# On the use of the "look-locker correction" for calculating T1 values from MOLLI

**DOI:** 10.1186/1532-429X-16-S1-P55

**Published:** 2014-01-16

**Authors:** Glenn S Slavin

**Affiliations:** 1 GE Healthcare, Bethesda, Maryland, USA

## Background

MOLLI [1] uses interleaved Look-Locker (LL) blocks for cardiac T1 mapping. Data is fit to the equation **A**-**B**exp(-TI/T1*) to yield an "apparent" T1 (T1*), which is dependent on both the true T1 and imaging parameters. To estimate true T1, a "LL correction" T1_est_=(**B**/**A**-1)T1* [Eq. 1] has been proposed [1,2]. Although this correction can provide reasonable estimates of true T1, we are not aware of a rigorous justification for its use. The purpose of this work was to investigate the applicability of this correction for MOLLI.

## Methods

**Figure 1:**
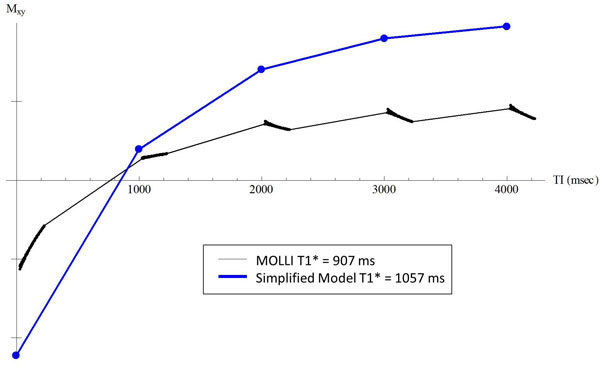
**Simulated recovery curves for i) MOLLI (single LL block) (black) and ii) simplified MOLLI model where the bSSFP readout is replaced by a single excitation (blue)**. Curves begin at different levels of transverse magnetization due to 8 dummy TRs used for MOLLI. Because the simplified model ignores the effects of multiple excitations, the recovery is dominated by free T1 relaxation. Thus, the T1* of the actual MOLLI data will always be less than that of the simplified model. T1_est _will be similarly underestimated. Simulated parameters: T1/T2 = 1200/40 ms (normal myocardium), TR = 3.0 ms, 35° flip angle; 60 bpm heart rate

**Figure 2:**
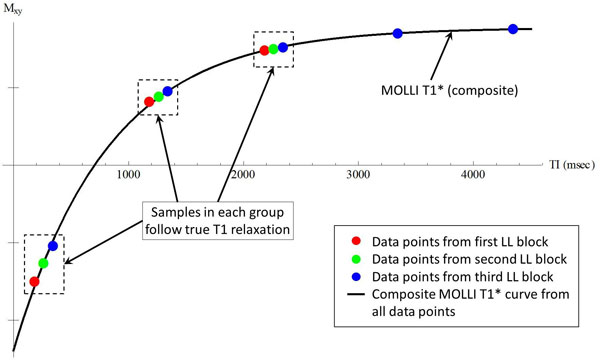
**Simulated magnetization recovery for MOLLI consisting of three interleaved LL blocks (T1 = 1200 ms)**. The composite MOLLI curve (black line) is made up of two distinct recovery regions: 1) Data points from individual LL blocks (red, green, and blue) which follow their respective (i.e., different) T1* and 2) data points in each hashed box which are essentially acquired in a single-point fashion and follow true T1 relaxation. The T1 contribution causes the composite T1* to be greater than the T1*s of the individual LL blocks. This counterintuitive result is in contrast to the mathematical expectation that a composite exponential curve should have a time constant intermediate to those of the component exponentials.

The LL correction (Eq. 1) is based on the following conditions [2]: 1) continuous imaging; 2) spoiled-gradient-echo (SPGR) readout; 3) TR << T1; and 4) negligible initial delays after inversion (TI_0_) and between images (e.g., IR cine). MOLLI uses a bSSFP readout which does not satisfy conditions 1 and 2. If the entire bSSFP readout were conceptually replaced with a single excitation pulse [3], MOLLI would approximate a LL-SPGR acquisition. However, this is not a theoretically valid simplification, and both T1* and T1_est _will be underestimated (Figure 1). This simplification also implies TR = T_RR _(RR interval). For cardiac imaging (T1≈1200 ms, T_RR _≈1000 ms), this violates condition 3 and can also be shown to underestimate T1_est_. Further, MOLLI utilizes non-zero TI_0_s which violate condition 4. As TI_0 _increases, the curve-fitting algorithm appropriately produces an increasingly negative y-intercept **A**-**B **(thus larger **B**/**A**) and a longer T1* (because of the added true T1 relaxation during TI_0_). Eq. 1 thus causes T1_est _to illogically become a function of TI_0 _which leads to overestimation at longer TI_0_. Finally, MOLLI involves the interleaving of three LL blocks, each with an incremented TI_0_. The resulting composite curve consists of two distinct regions of magnetization recovery (Figure 2). This has the counterintuitive effect of causing the composite T1* to be larger than the individual T1*s.

## Results

The MOLLI acquisition does not satisfy the requirements on which the LL correction is based. For a single LL block, each violation produces an error in T1_est_. When LL blocks are combined, however, the overestimation caused by interleaving LL blocks obtained with non-zero TI_0 _partially offsets the underestimation from the misapplied simplification and correction. Under certain conditions, this yields a reasonable estimate of T1, with the error being strongly dependent on the range of TI_0_. In practice, TI_0 _is typically too short to completely offset the effects of the LL correction, resulting in the observed systematic underestimation of T1.

## Conclusions

The use of multiple LL blocks in MOLLI was intended to improve accuracy by increasing the sampling of the relaxation curve. Instead, it can be shown that this distinguishing feature of MOLLI has the unexpected effect of essentially averaging out errors introduced by the LL correction. However, T1 estimates derived from MOLLI using the LL correction cannot be consistently accurate because of the violated conditions of its use.

## Funding

None.
